# Integrated Aroma Profiling and Functional Characterization of LcTPS24 Uncover Its Role in Monoterpene Biosynthesis in Litchi Fruit

**DOI:** 10.3390/plants15213256

**Published:** 2026-10-22

**Authors:** Farhat Abbas, Xibing Deng, Jing Wu, Tingting Yan, Mingchao Yang, Zhe Chen, Xianghe Wang, Fuchu Hu, Hui-Cong Wang

**Affiliations:** 1 Key Laboratory of Biology and Genetic Improvement of Horticultural Crops-South China/Guangdong Litchi Engineering Research Center, College of Horticulture, South China Agricultural University, Guangzhou 510640, China; 2 Institute of Tropical Fruit Trees, Hainan Academy of Agricultural Sciences, Haikou 571100, China; 3 Key Laboratory of Genetic Resources and Utilization of Tropical Fruits and Vegetables (Co-Construction by Ministry and Province), Ministry of Agriculture and Rural Affairs, Haikou 571100, China; 4 Key Laboratory of Tropical Fruit Tree Biology of Hainan Province, Haikou 571100, China; 5 Sanya Research Institute, Hainan Academy of Agricultural Sciences, Sanya 572025, China

**Keywords:** *Litchi chinensis*, fruit aroma, terpene synthase, GC-MS, LcTPS24

## Abstract

Litchi (*Litchi chinensis*) is a commercially valuable subtropical fruit prized for its unique floral–fruity aroma, which largely determines consumer preference and commercial quality. Terpenoids serve as the core volatile compounds that shape the characteristic litchi fruit aroma. Despite their importance, the volatile profile of litchi fruit and the key genes underlying its aroma formation remain poorly understood. Herein, we determine the gas chromatography–mass spectrometry (GC-MS)-based metabolomic profiling of litchi cultivars and functionally characterize a key litchi terpene synthase gene, LcTPS24, a candidate selected on the basis of previously reported TPS-family expression patterns and gene–metabolite associations. GC-MS profiling of four commercially significant cultivars, i.e., Feicui (FC), Guiwei (GW), Jingganghongnuo (JGHN), and Lufennuo (LFN), at three developmental stages revealed that terpenes predominate the volatile organic compound (VOC) spectrum, accounting for up to 94% of total volatiles in JGHN. Monoterpenes represent the principal fraction in GW (98%), JGHN (81%), and FC (75%), while comprising 44% in the LFN cultivar. Phylogenetic analysis classified LcTPS24 within the TPS-b subfamily of angiosperm monoterpene synthases. Biochemical assays performed through prokaryotic expression and HS-SPME-GC-MS revealed that recombinant LcTPS24 exclusively utilizes geranyl pyrophosphate (GPP) rather than farnesyl pyrophosphate (FPP), producing ten monoterpene products: α-thujene, α-pinene, (+)-sabinene, (−)-β-pinene, α-phellandrene, α-terpinene, 2-carene, p-cymene, γ-terpinene, and (+)-4-carene. In JGHN fruit, virus-induced gene silencing was associated with reduced *LcTPS24* transcript abundance and lower monoterpene accumulation, providing evidence consistent with a contribution to fruit monoterpene biosynthesis. Electronic-tongue measurements were analyzed separately to examine treatment-associated variation in juice sensor responses, although these measurements did not directly assess volatile composition or aroma perception. This study functionally validates the role of LcTPS24 in terpenoid volatile biosynthesis and provides genetic resources and mechanistic insights for molecular breeding of high-aroma litchi cultivars.

## 1. Introduction

Fruit aroma serves as a core determinant of commercial quality, profoundly shaping consumer sensory perception and market purchasing preferences for fresh horticultural products. Fruit aroma arises from complex mixtures of volatile organic compounds (VOCs), including terpenoids, esters, and aldehydes, whose abundance and sensory properties contribute to the distinctive characteristics of individual fruits and influence consumer preference [1,2,3]. These volatile metabolites interact dynamically with olfactory receptors in vivo, collectively modulating flavor perception and delineating the distinct sensory attributes of fruit [3,4,5]. Several studies have confirmed that qualitative and quantitative variations in terpene VOCs are the main cause of intra-species flavor differences in woody fruit crops, making terpene metabolic regulation a central focus of fruit flavor molecular research [6,7,8]. Elucidating the genetic basis of terpene metabolism is critical for dissecting mechanisms of flavor formation and supporting precision flavor breeding in perennial fruit species.

Litchi (*Litchi chinensis* Sonn.), a highly profitable subtropical fruit species in the Sapindaceae family, is extensively cultivated in South and Southeast Asia. It is uniquely characterized by its distinctive aromatic fragrance, a key commercial trait that sustains its high premium market [9]. Litchi is extensively cultivated in China, India, Vietnam, and Thailand, with global production surpassing 2.5 million metric tons per year [10]. The quality of the fruit, especially its aroma, is a decisive factor influencing cultivar selection and commercial export values. Its volatile profile includes both monoterpenes and sesquiterpenes, and their accumulation varies among cultivars and throughout fruit development [11,12]. Characterizing the enzymes involved in producing these compounds is therefore necessary to clarify how terpene biosynthesis contributes to the observed variation in litchi fruit volatile composition.

Terpene biosynthesis depends on the availability of prenyl diphosphate substrates and the catalytic activities of terpene synthases (TPSs), with monoterpenes generally synthesized from geranyl diphosphate (GPP) and sesquiterpenes from farnesyl diphosphate (FPP). The precursors for these reactions are supplied by the plastidial methylerythritol phosphate (MEP) pathway and the predominantly cytosolic mevalonate (MVA) pathway, although exchange between cellular compartments can modify the relationship between precursor origin and the site of product formation. Monoterpenes such as α-pinene, β-pinene, sabinene, linalool, β-ocimene, limonene, and related C_10_ hydrocarbons contribute green, woody, and spicy olfactory notes that form the structural backdrop of litchi fragrance [12,13,14]. Consequently, identification of a terpene product class alone is insufficient to establish the biosynthetic pathway or subcellular compartment responsible for its synthesis [15,16].

Plant TPSs generate structurally diverse products from prenyl diphosphate substrates, and individual enzymes may catalyze the formation of several compounds from the same substrate [17]. Although sequence similarity and expression patterns can guide the selection of candidate genes, biochemical assays are required to establish their catalytic activities, while complementary genetic experiments help evaluate their contributions in planta. Previous studies have characterized the litchi TPS family and the activities of individual enzymes [11,12,18]; nevertheless, the contributions of additional TPSs to developmental and cultivar-dependent variation in fruit volatiles remain in-completely understood.

Our previous genome-wide analysis identified 38 litchi TPS genes and characterized their sequence features and expression patterns, providing a basis for selecting candidates for functional investigation [11]. Among these genes, *LcTPS24* (LI-TCHI028835.m1) exhibited elevated expression during early pericarp development, while integrated transcriptomic and metabolomic analyses revealed positive associations with limonene and terpinolene and negative associations with several sesquiterpenes. These observations justified prioritizing LcTPS24 for further study, although they did not establish that the associated compounds were its direct products or that it contributed more strongly than other TPS genes to fruit aroma. Accordingly, the present study combined VOC profiling across cultivars and developmental stages with expression analysis, recombinant enzyme assays, and virus-induced gene silencing (VIGS) to investigate the biochemical activity of LcTPS24 and its contribution to monoterpene accumulation in litchi fruit.

## 2. Materials and Methods

### 2.1. Plant Materials and Fruit Sampling

Four litchi cultivars with significantly divergent aroma characteristics, namely Feicui (FC), Guiwei (GW), Jingganghongnuo (JGHN), and Lufennuo (LFN), were selected as experimental materials from a commercial orchard in Conghua county, GuangZhou, Guangdong Province, China. All litchi trees were cultivated under consistent field management regimes to eliminate environmental and agronomic interference. To systematically characterize the dynamic accumulation patterns of aromatic volatiles during fruit development, litchi fruits were collected at three representative developmental stages: S1 (immature green fruit stage), S2 (color transition and middle ripening stage), and S3 (full commercial maturation stage). After harvest, the peel (pericarp) and pulp (aril) tissues were rapidly separated via manual dissection. All tissue samples were immediately snap-frozen in liquid nitrogen and preserved at −80 °C for subsequent biochemical and molecular analyses. For each cultivar and each developmental stage, at least three independent biological replicates were prepared, with each replicate consisting of mixed fruit samples collected from no fewer than three healthy litchi trees.

### 2.2. Volatile Organic Compound Profiling

Volatile organic compounds (VOCs) were extracted from litchi peel and pulp tissue using headspace solid-phase microextraction (HS-SPME) as explained earlier [19]. Briefly, 2.0 g of freshly homogenized sample was transferred to a sealed 20 mL glass headspace vial containing 1 g of decanoic acid as an internal standard. Samples were thermally equilibrated at 40 °C for 30 min, after which a preconditioned 50/30 μm divinylbenzene/carboxen/polydimethylsiloxane (DVB/CAR/PDMS) SPME fiber (Supelco, Bellefonte, PA, USA) was subjected to the headspace for an additional 30 min to achieve adsorption of volatile analytes. Thermal desorption and chromatographic separation were performed on an Agilent 7890B gas chromatograph interfaced with a 5977B single quadrupole mass selective detector (Agilent Technologies, Santa Clara, CA, USA) as described previously [11,19,20]. Analyte separation was achieved on an HP-5MS fused-silica capillary column (30 m × 0.25 mm i.d. × 0.25 μm film thickness) under optimized temperature programming conditions.

### 2.3. GC-MS Condition and Volatile Identification

The GCMS system was equipped with an Agilent HP-5MS capillary column (30 m, 0.25 mm), and helium gas was used as a carrier at a constant flow rate of 1 mL/min. Helium was used as the carrier gas at a constant flow rate of 1 mL min^−1^. The oven temperature was maintained at 40 °C for 2 min, increased to 250 °C at 5 °C min^−1^, and held at 250 °C for 5 min. The quantification of volatiles was measured using the Agilent ChemStation Data Analysis Application (ChemStation version C) based on the peak areas and quantity of internal standard. The cited identification protocol incorporates linear retention indices calculated using C7–C40 n-alkanes and comparisons of retention times. A comparison of mass spectra with NIST 14 at a match factor around 80 was used to determine the identity of various substances found in the samples. The chromatographic running time was 30 min and three to four replicates were tested. Relative volatile composition was expressed as the percentage contribution of each compound to the total integrated peak area. Ethyl caprate (1.728 μg per sample) was used as an internal standard at a fixed concentration prior to extraction.

### 2.4. Electronic Tongue (E-Tongue) Flavor Profile Analysis

Overall flavor profile discrimination between *LcTPS24*-silenced (pTRV2-*LcTPS24*) and control (pTRV2-empty) litchi fruits was assessed using an α-ASTREE electronic tongue liquid analyzer (Alpha MOS, Toulouse, France). This system employs an array of seven cross-selective potentiometric electrochemical sensors, each with a distinct chemical sensitivity profile, enabling simultaneous multidimensional characterization of the liquid taste matrix without the need for trained sensory panels. Before analysis, fresh aril juice was extracted from harvested fruits by gentle manual pressing and clarified by filtration through a 0.45 μm membrane. It was diluted ten-fold (*v*/*v*) with ultrapure water (18.2 MΩ·cm) to bring analyte concentrations within the linear response range of the sensor array. Each sample was subjected to three independent technical replicates, with a 120 s signal acquisition window per sensor per replicate to ensure equilibration and a stable electrochemical response. Raw multi-sensor response vectors were exported and subjected to principal component analysis (PCA) using Alpha Soft v.12 analytical software (Alpha MOS) to reduce the dimensionality of the sensor response space and visualize the degree of flavor profile separation between experimental groups. The discriminant index (DI), derived from PCA, was used as a quantitative measure of the overall discriminatory power between the pTRV2-*LcTPS24* and pTRV2-empty treatment groups.

### 2.5. Quantitative Real-Time PCR (qRT-PCR)

Total RNA was extracted from frozen litchi tissues using the CTAB-LiCl precipitation protocol, followed by DNase I treatment and column purification (RNeasy Plant Mini Kit; Qiagen, Venlo, The Netherlands), as described previously [11]. First-strand cDNA was synthesized from 1 μg total RNA using the PrimeScript RT reagent kit with gDNA Eraser (TaKaRa, Shiga, Japan). qRT-PCR was conducted on a CFX96 Touch system (Bio-Rad) using TB Green Premix Ex Taq II (TaKaRa, Shiga, Japan). Litchi Actin served as the reference gene for normalization. Relative expression was calculated by the 2^−ΔΔCt^ method [21] from three biological replicates. Expression heatmaps were generated using TBtools v2.0 [22].

### 2.6. Recombinant Protein Expression and In Vitro Enzyme Assay

The predicted chloroplast transit peptide of LcTPS24 was identified using TargetP-2.0 and excluded from the expression construct as explained earlier [19]. The truncated coding sequence was cloned into pET-28a (Novagen, Darmstadt, Germany) and transformed into *Escherichia coli* BL21(DE3). Recombinant His-tagged LcTPS24 protein expression was induced with 0.5 mM IPTG at 16 °C for 16 h and purified by Ni-NTA affinity chromatography (Qiagen). Enzyme assays were performed by incubating 50 μg of purified protein with 100 μM GPP or FPP (Sigma-Aldrich, St. Louis, MO, USA) in assay buffer (50 mM HEPES, pH 7.2, 10 mM MgCl_2_, 5 mM DTT, 5% glycerol) at 30 °C for 60 min. Products were extracted by pentane overlay and analyzed by GC-MS with empty vector-expressed protein served as the negative control.

### 2.7. Virus-Induced Gene Silencing (VIGS)

Virus-induced gene silencing (VIGS) was carried out as explained earlier [23]. In short, A 300 bp *LcTPS24*-specific fragment (selected to minimize off-target effects by BlastN screening against the litchi transcriptome) was cloned into the pTRV2 vector as previously described [24]. *Agrobacterium tumefaciens* GV3101 suspensions harboring pTRV1 and pTRV2-*LcTPS24* or pTRV2-empty were mixed 1:1 (OD_600_ ≈ 1.0) and co-infiltrated into the calyx end of S1-stage litchi fruitlets using a needleless syringe in cultivar JGHN. Infiltrated fruits were harvested approximately 21 days post-infiltration. The empty vectors pTRV2 and pTRV1 were used for control. Ten to twenty fruit clusters were treated in each tree at about two weeks before harvest. The assay was performed in triplicate as explained above.

### 2.8. Statistical Analysis

All data are presented as mean ± SE of ≥3 independent biological replicates. Least Significant Difference (LSD) procedure following a significant one-way ANOVA was applied in multiple factor assays. Difference letters above the bars indicate significant difference at *p* < 0.05. *t*-test (two-tailed) was used for pairwise comparisons between pTRV2-empty and pTRV2-LcTPS24. * and ** indicate significant difference at *p* < 0.05 and *p* < 0.01, respectively. PCA and heatmap visualizations were conducted in Tbtools.

## 3. Results

### 3.1. VOC Profile Across Litchi Cultivars and Ripening Stages

**Figure 1:**
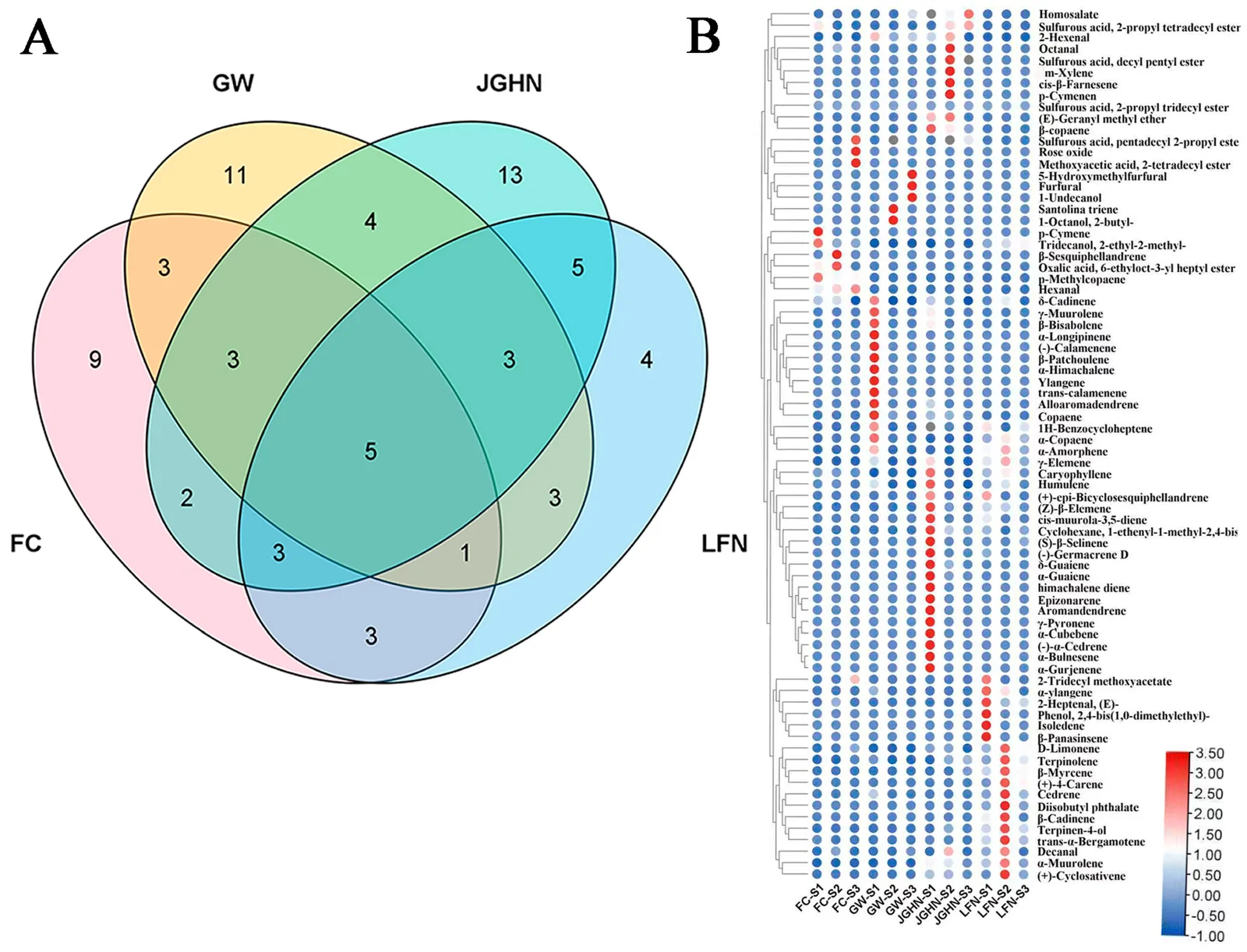
Venn diagram and Heatmap of VOCs identified from litchi fruit. (**A**) Shared and cultivar-specific VOC detections in FC, GW, JGHN, and LFN. (**B**) Heatmap of VOC abundance across cultivar–stage combinations (S1–S3), with hierarchical clustering of compounds; red and blue indicate higher and lower relative abundance, respectively.

To characterize the volatile profiles of litchi fruit, volatile organic compounds (VOCs) were analyzed in combined peel and pulp samples from four cultivars (FC, GW, JGHN, and LFN) at three developmental stages (S1, S2, and S3) using HS-SPME–GC–MS (Figure 1). JGHN exhibited the greatest number of detected compounds (45 VOCs), followed by LFN (33), GW (30), and FC (23) (Figure 1A). Venn diagram analysis identified five VOCs—humulene, terpinolene, decanal, caryophyllene, and δ-cadinene—that were shared among all four cultivars, together with compounds detected exclusively in individual cultivars, indicating variation in volatile composition (Figure 1A). Compounds reported as unique to JGHN included α/β-guaiene, himachalene diene, α-copaene, α-cubebene, α-bulnesene, sulfurous acid decyl pentyl ester, (−)-α-cedrene, cis-β-farnesene, epizonarene, m-xylene, α-gurjenene, aromadendrene, and p-cymene. Those reported as unique to GW included β-patchoulene, α-himachalene, furfural, (+)-4-carene, 1-undecanol, 5-hydroxymethylfurfural, (−)-calamenene, ylangene, β-longipinene, trans-calamenene, and 2-butyl-1-octanol. Compounds reported as unique to FC included santolina triene, δ-pyronene, p-cymene, β-sesquiphellandrene, oxalic acid 6-ethyloct-3-yl heptyl ester, (E)-geranyl methyl ether, rose oxide, p-methylcopaene, and methoxyacetic acid 2-tetradecyl ester. LFN exhibited the fewest reported cultivar-specific compounds, comprising β-panasinsene, diisobutyl phthalate, isoledene, and 2,4-di-tert-butylphenol. Heatmap analysis further revealed distinct patterns of VOC accumulation across cultivars and developmental stages (Figure 1B).

### 3.2. Terpenes Are the Predominant Volatile Class Across Litchi Cultivars and Ripening Stages

**Figure 2:**
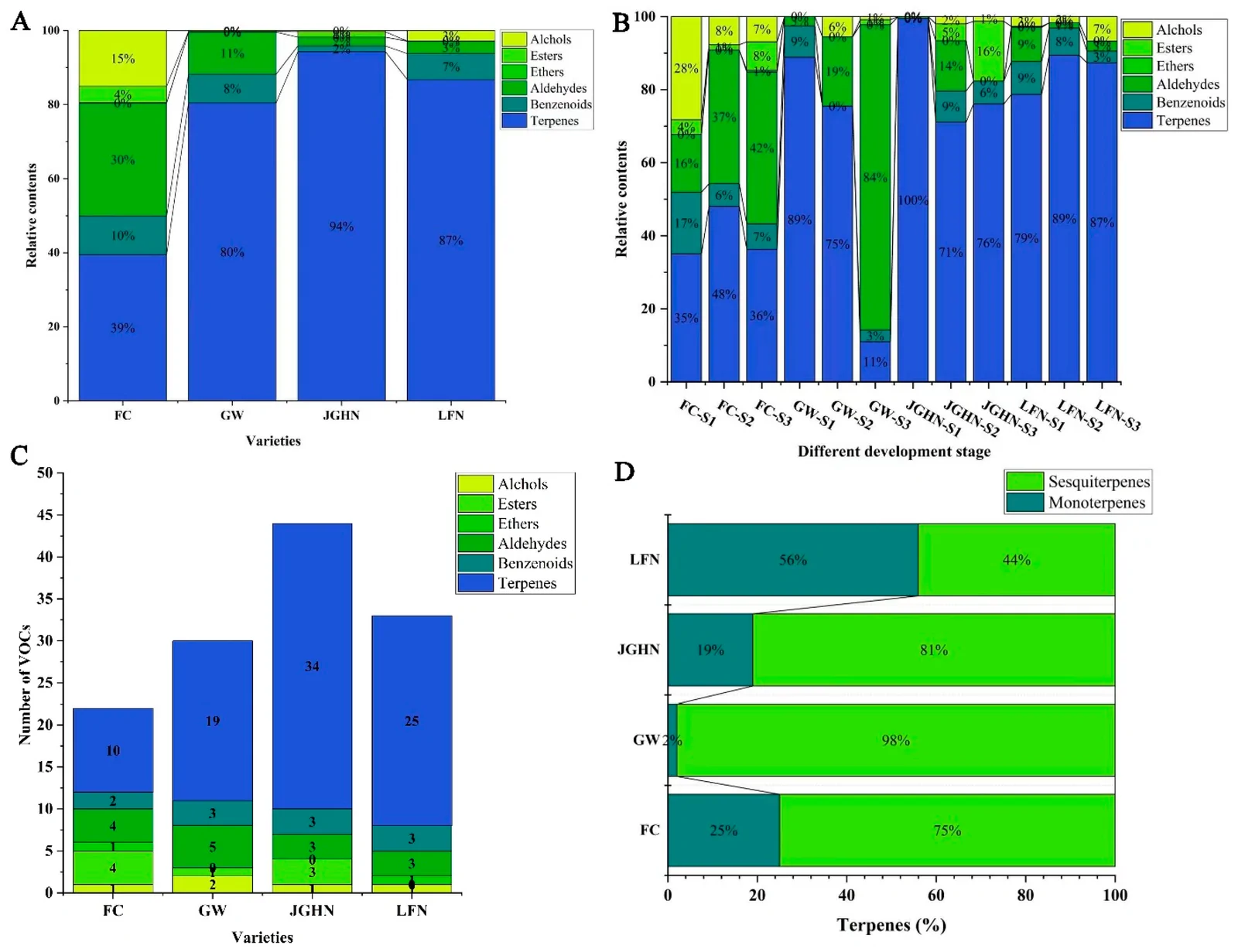
**Volatile terpene profiles differ among four litchi cultivars and across fruit developmental stages**. (**A**) Relative content (%) of six volatile organic compound (VOC) classes, i.e., alcohols, esters, ethers, aldehydes, benzenoids, and terpenes, in the four cultivars FC, GW, JGHN, and LFN, showing terpenes as the dominant class in all cultivars (39–94%). (**B**) Relative content (%) of the same VOC classes at three developmental stages (S1–S3) for each cultivar, showing a progressive shift toward terpene dominance as fruit ripens. (**C**) Total number of VOCs detected within each chemical class for the four cultivars. (**D**) Proportion of monoterpenes versus sesquiterpenes among total terpenes in each cultivar, highlighting cultivar-specific differences in terpene subclass composition.

Six chemical classes—alcohols, esters, ethers, aldehydes, benzenoids, and terpenes—were detected in combined peel and aril samples, with their relative contributions varying among cultivars and developmental stages (Figure 2A,B). In the cultivar-level summaries across S1–S3, terpenes represented the largest chemical class, accounting for approximately 39% of total VOC abundance in FC, 80% in GW, 94% in JGHN, and 87% in LFN (Figure 2A). Aldehydes contributed approximately 30%, 11%, 2%, and 3% in FC, GW, JGHN, and LFN, respectively. Alcohols accounted for approximately 15% in FC and 3% in LFN but were not detected in GW or JGHN, whereas benzenoids contributed approximately 10%, 8%, 2%, and 7% in FC, GW, JGHN, and LFN, respectively.

Stage-specific profiles revealed developmental shifts in volatile composition, including changes in the relative contributions of terpenes, aldehydes, alcohols, and esters (Figure 2B). In GW, terpenes predominated during S1 and S2, accounting for up to approximately 89% of total VOC abundance, whereas aldehydes became the predominant class at S3, contributing approximately 84%. In JGHN, terpenes accounted for approximately 100% of the recorded VOC abundance at S1, declining to 71% at S2 and increasing slightly to 76% at S3.

Remarkably, the quantity of terpenes significantly exceeded that of other types of volatile organic compounds (VOCs). The highest quantity of terpene volatile diversity was observed in JGHN (34), followed by LFN (25) and GW (19), while the lowest was in FC (10) (Figure 2C). The quantities of benzenoids and aldehydes were similar among all cultivars. Ether, esters, and alcohols were among the least found compounds across all cultivars. An analysis of the terpene fraction disclosed notable cultivar-specific variations in the monoterpene-to-sesquiterpene ratio: GW was predominantly monoterpenoid (98% monoterpenes) (Figure 2D). In LFN, sesquiterpenes constituted the larger proportion of the terpene fraction, accounting for approximately 56%.

### 3.3. Characterization of LcTPS24

Our previous analysis of the litchi TPS family identified LcTPS24 as a candidate associated with volatile terpene metabolism, based on its elevated expression during early pericarp development and its associations with monoterpenes in the integrated transcriptomic and metabolomic analysis. These findings provided the rationale for investigating its biochemical activity and contribution to fruit terpene biosynthesis in the present study.

**Figure 3:**
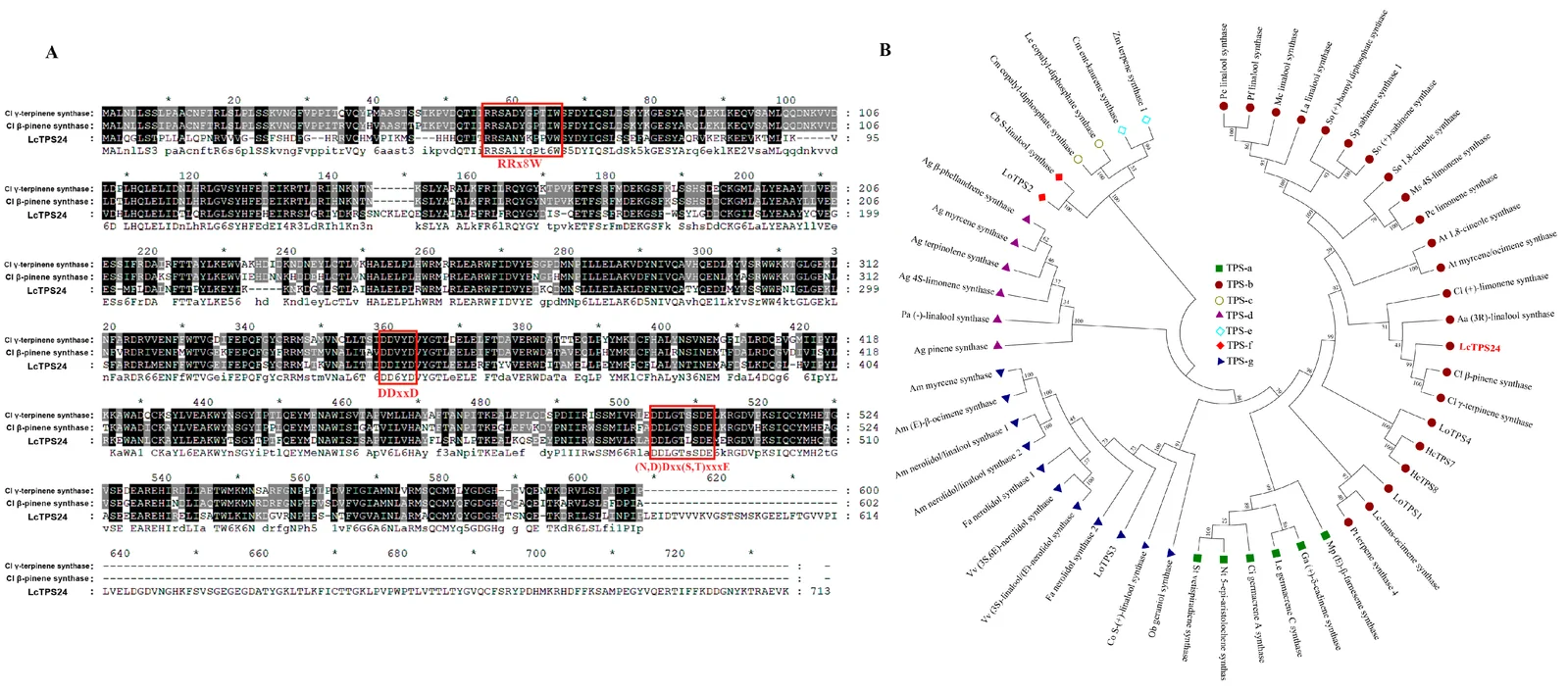
**Sequence and phylogenetic analysis of LcTPS24**. (**A**) Multiple sequence alignment of LcTPS24 with *Citrus limon* γ-terpinene synthase and β-pinene synthase. Conserved terpene synthase motifs—RRx_8_W, DDxD, and (N,D)Dxx (S,T)xxxE—are boxed in red; black and gray shading indicate identical and similar residues, respectively. (**B**) Phylogenetic tree of LcTPS24 and terpene synthases from other plant species, constructed to classify LcTPS24 within the TPS gene family. Colored/shaped symbols denote TPS subfamilies (TPS-a through TPS-g); LcTPS24 (highlighted in red text) clusters within the TPS-b subfamily. Numbers at nodes indicate bootstrap support values.

Multiple sequence alignment of LcTPS24 with γ-terpinene synthase and β-pinene synthase from *Citrus* revealed conserved sequence features characteristic of plant terpene synthases, including the N-terminal RRx_8_W motif and the C-terminal DDxxD and NSE/DTE motifs (Figure 3A). The latter two motifs are associated with metal-ion coordination and metal-dependent ionization of prenyl diphosphate substrates. Their presence supports the annotation of LcTPS24 as a putative terpene synthase, although conservation of these motifs alone does not establish catalytic activity or substrate specificity. In the phylogenetic tree presented in Figure 3B, LcTPS24 clustered within the TPS-b subfamily, which includes many angiosperm monoterpene synthases. Together, the sequence features and phylogenetic placement provided a basis for investigating its proposed monoterpene synthase activity through biochemical assays.

### 3.4. LcTPS24 Is Primarily Expressed in the Peel, Exhibiting Cultivar- and Stage-Specific Regulation

**Figure 4:**
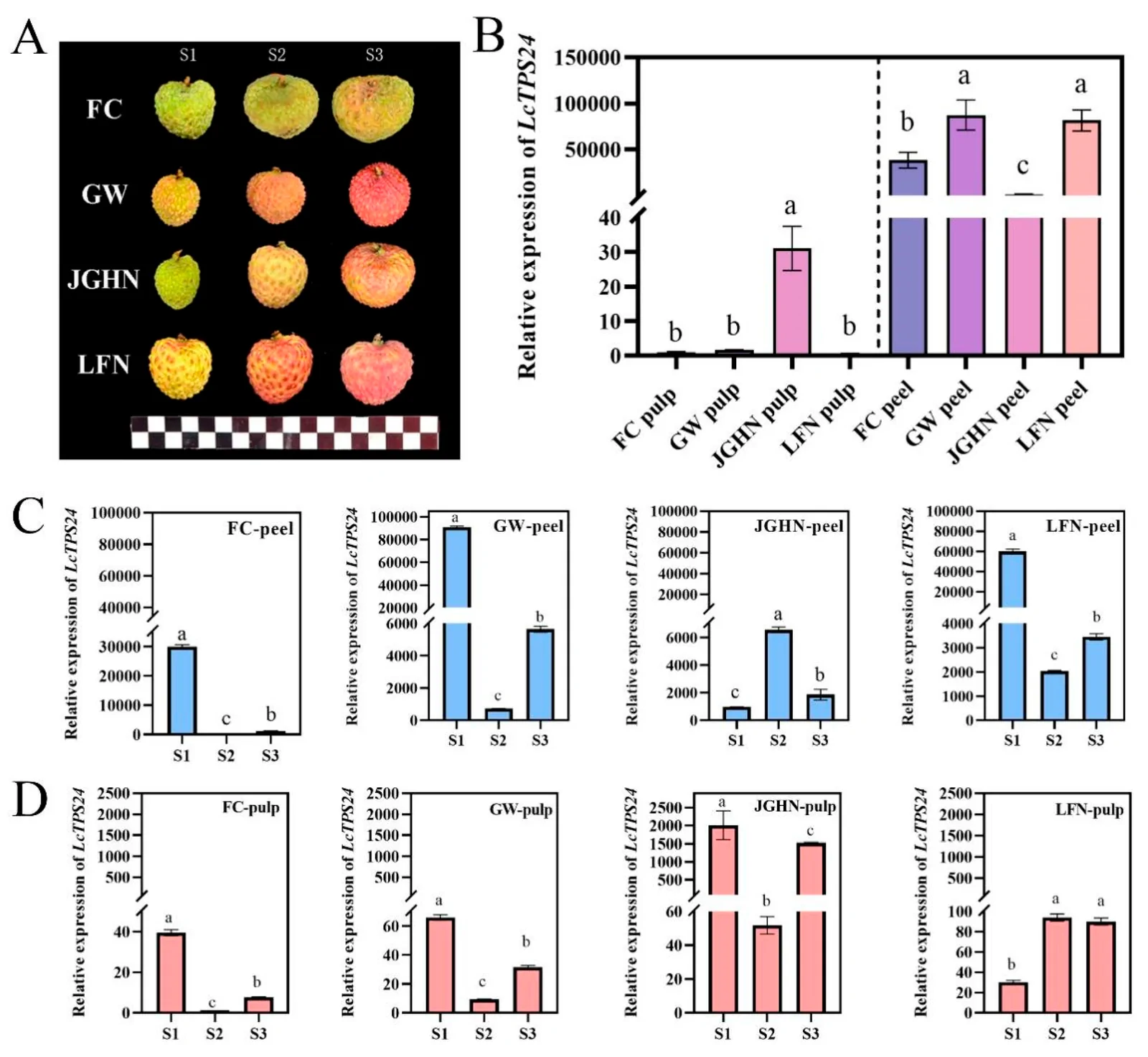
***LcTPS24* expression across cultivars and developmental stages.** (**A**) Representative images of fruit from cultivars FC, GW, JGHN, and LFN at three developmental stages (S1–S3), illustrating progressive peel color change during ripening. Scale bar indicated by checkered ruler. (**B**) Relative expression of *LcTPS24* in pulp and peel tissues of the four cultivars. Different lowercase letters indicate statistically significant differences (LSD, *p* < 0.05). (**C**) Relative expression of *LcTPS24* in peel tissue across developmental stages (S1–S3) for each cultivar. (**D**) Relative expression of *LcTPS24* in pulp tissue across developmental stages (S1–S3) for each cultivar. Different lowercase letters indicate statistically significant differences among stages within each cultivar (LSD, *p* < 0.05). Data are presented as the mean ± SEM (*n* = 3).

To investigate the potential relationship between *LcTPS24* expression and fruit volatile production, its spatial and temporal expression patterns were examined in the peel and aril of four litchi cultivars across three successive developmental stages (Figure 4). RT–qPCR analysis revealed variation in transcript abundance among tissues, cultivars, and developmental stages, providing an expression-based context for investigating the contribution of LcTPS24 to fruit terpene biosynthesis. The sampled developmental stages are illustrated in Figure 4A, and the tissue comparison is presented in Figure 4B. Across all four cultivars, the plotted expression values were higher in the peel than in the aril. Cultivar-specific comparisons revealed contrasting expression patterns between the two tissues: JGHN exhibited the highest aril expression, whereas peel expression was highest in GW, followed by LFN, FC, and JGHN.

During peel development, *LcTPS24* transcript abundance reached its highest level at S1 in FC, GW, and LFN, whereas the highest expression in JGHN occurred at S2 (Figure 4C). In GW and LFN, expression decreased from S1 to S2 and subsequently increased at S3, although the S3 values remained below those observed at S1. FC exhibited a different developmental trajectory, with a pronounced decline after its S1 expression peak and comparatively low transcript abundance at the subsequent stages. In JGHN, expression increased from S1 to S2 before declining at S3, indicating that its peel expression maximum occurred later than in the other three cultivars.

In the aril, FC, GW, and JGHN exhibited broadly similar developmental expression patterns, characterized by the highest transcript abundance at S1, a decrease at S2, and a partial recovery at S3 (Figure 4D). By contrast, LFN exhibited lower expression at S1, followed by an increase at S2 and a broadly comparable expression level at S3. These profiles indicate that the developmental pattern of *LcTPS24* expression differed between tissues and among cultivars, particularly in the timing of maximum transcript accumulation. Although JGHN showed the highest expression in the aril comparison, this ranking did not extend to the peel, where GW exhibited the highest expression. Collectively, these observations provide a basis for examining the relationship between LcTPS24 expression and terpene accumulation, while expression patterns alone do not establish a direct quantitative relationship with volatile production.

### 3.5. Recombinant LcTPS24 Functions as a GPP-Dependent Multi-Product Monoterpene Synthase

**Figure 5:**
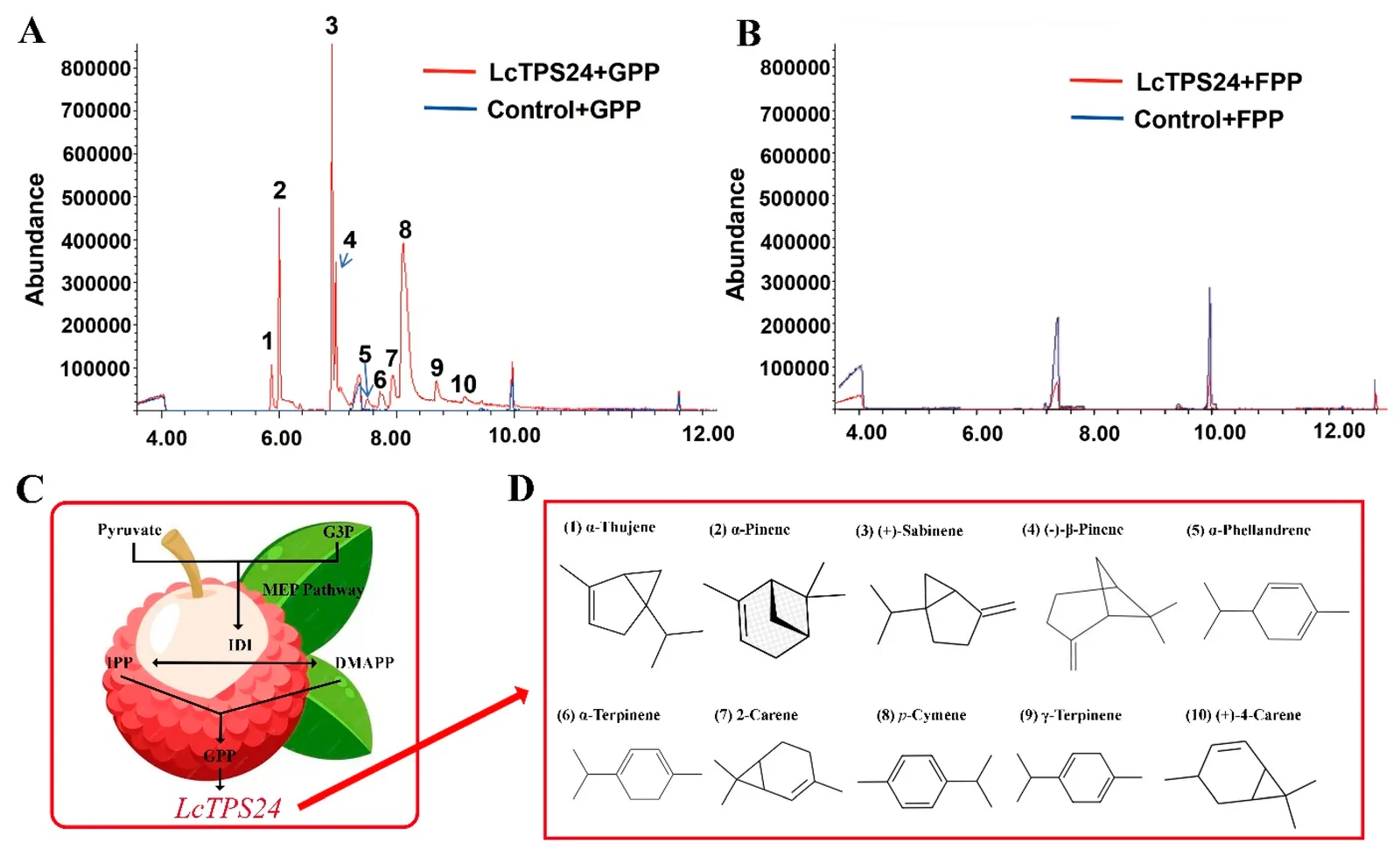
**Functional characterization of LcTPS24 as a monoterpene synthase.** (**A**) GC-MS chromatogram of in vitro enzymatic assay products from recombinant LcTPS24 protein incubated with the substrate geranyl pyrophosphate (GPP) (red trace), compared with a control reaction lacking functional enzyme (blue trace). Numbered peaks correspond to the ten monoterpene products identified. (**B**) Chromatogram of enzymatic activity of LcTPS24 protein incubated with the substrate farnesyl pyrophosphate (FPP). (**C**) Schematic of the methylerythritol phosphate (MEP) pathway in litchi fruit, showing the conversion of pyruvate and glyceraldehyde-3-phosphate (G3P) into isopentenyl diphosphate (IPP) and dimethylallyl diphosphate (DMAPP) via IDI, and the subsequent formation of GPP, the substrate used by LcTPS24. (**D**) Chemical structures of the ten monoterpene products generated by LcTPS24 from GPP: (**1**) α-thujene, (**2**) α-pinene, (**3**) (+)-sabinene, (**4**) (−)-β-pinene, (**5**) α-phellandrene, (**6**) α-terpinene, (**7**) 2-carene, (**8**) p-cymene, (**9**) γ-terpinene, and (**10**) (+)-4-carene.

To characterize the enzymatic activity of LcTPS24, a His-tagged recombinant protein was expressed in *Escherichia coli* and subjected to in vitro assays using geranyl diphosphate (GPP) and farnesyl diphosphate (FPP) as substrates. GC–MS analysis of the GPP reaction revealed ten peaks tentatively assigned to monoterpene products: (1) α-thujene, (2) α-pinene, (3) sabinene, (4) β-pinene, (5) α-phellandrene, (6) α-terpinene, (7) 2-carene, (8) p-cymene, (9) γ-terpinene, and (10) 4-carene (Figure 5A). Based on the reported integrated peak-area proportions, p-cymene was the most abundant product, accounting for 40.3% of the summed product peak area, followed by sabinene (23.2%), α-pinene (11.9%), and β-pinene (8.3%). The remaining products comprised α-thujene (4.7%), α-terpinene (4.4%), γ-terpinene (3.1%), and α-phellandrene (2.6%), while 4-carene and 2-carene together accounted for approximately 0.6%. These proportions describe the relative chromatographic contributions of the assigned products rather than their absolute concentrations or the catalytic efficiency of LcTPS24.

No corresponding products were detected in the negative-control reactions, and no LcTPS24-dependent product formation was detected when FPP was supplied under the assay conditions (Figure 5A,B). These findings support the characterization of LcTPS24 as a GPP-dependent, multiproduct monoterpene synthase under the conditions tested. Although this activity is consistent with a potential role in monoterpene biosynthesis associated with MEP-derived precursors, the enzyme assays alone do not establish its subcellular localization or the pathway supplying its substrate in planta. Figure 5C illustrates the proposed biosynthetic context, while Figure 5D presents the proposed chemical structures corresponding to the ten assigned products.

### 3.6. VIGS-Induced Silencing of LcTPS24 Reduces Terpene Levels and Modifies Fruit Flavor

**Figure 6:**
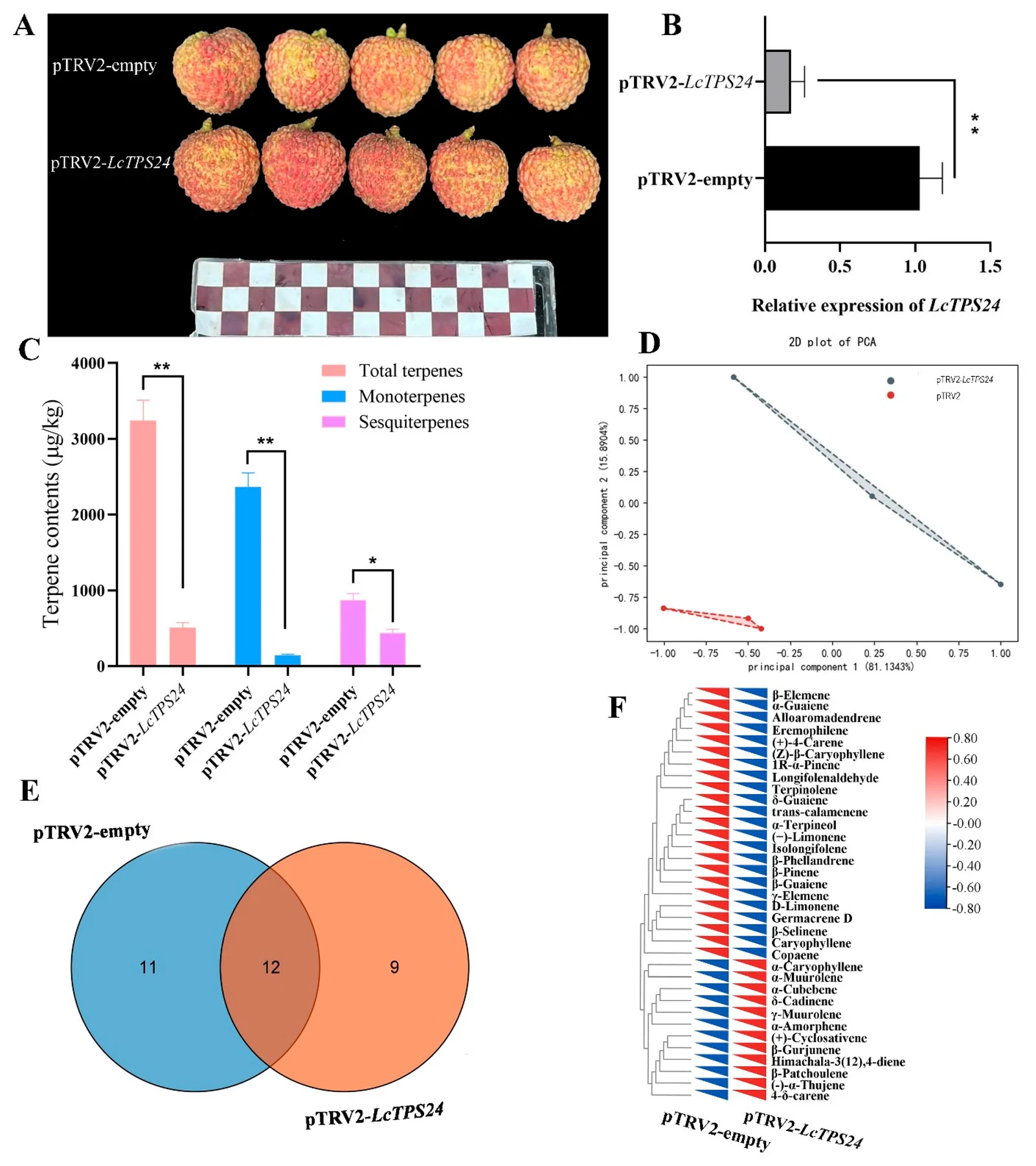
**Silencing of LcTPS24 alters fruit volatile profile**. (**A**) Representative images of litchi fruit infiltrated with empty vector control (pTRV2-empty) or LcTPS24-silencing construct (pTRV2-LcTPS24). Scale bar indicated by checkered ruler. (**B**) Relative expression of LcTPS24 in pTRV2-empty and pTRV2-LcTPS24 fruit, confirming silencing efficiency. Asterisk indicates a statistically significant difference (*t*-test, ** *p* < 0.01). (**C**) Total terpene, monoterpene, and sesquiterpene contents (μg/kg) in pTRV2-empty and pTRV2-*LcTPS24* fruit. Asterisks indicate statistically significant differences (*t*-test, * *p* < 0.05, ** *p* < 0.01). (**D**) Two-dimensional principal component analysis (PCA) plot of E-tingue based sensory profiles, showing separation between pTRV2-empty and pTRV2-*LcTPS24* samples along principal component 1 (81.13% variance explained) using the data from E-tongue. (**E**) Venn diagram showing the number of terpene compounds unique to and shared between pTRV2-empty and pTRV2-*LcTPS24* fruits. (**F**) Heatmap highlighting the number of VOCs emitted in *LcTPS24*-silenced and control fruits.

We employed VIGS using the TRV vector system [23,24] to verify the in planta function of *LcTPS24* in litchi monoterpene synthesis. The silenced (pTRV2-*LcTPS24*) and control (pTRV2-empty) fruits appeared uniform in appearance at the time of harvest (Figure 6A). qRT-PCR validated significant gene silencing, with LcTPS24 transcript levels considerably lower than in control fruits (Figure 6B). Further, we performed GC-MS using HS-SPME to assess the silencing effect of *LcTPS24* on fruit aroma (Figure 6C). GC-MS analysis revealed a substantial decrease in terpene levels in *LcTPS24*-silenced fruits compared with controls. The total terpene level decreased from nearly 3250 μg/kg in control fruits to about 530 μg/kg in silenced fruits, representing a reduction of more than 83%. The monoterpene content declined significantly, from almost 2370 to about 100 μg/kg, representing a loss of more than 95%. The sesquiterpene content decreased markedly, from roughly 900 to about 450 μg/kg.

To verify the silencing effect of *LcTPS24* on fruit aroma, we further performed E-tongue analysis (Figure 6D). The PCA of the volatile profiles confirmed total separation of the pTRV2-empty and pTRV2-*LcTPS24* groups along PC1 (81.13%) and PC2 (15.89%), hence affirming unique terpene compositions. Venn diagram analysis revealed that 11 terpene compounds [isolongifolene, β-pinene, (+)-4-carene, trans-calamenene, limonene, longifolenaldehyde, β-guaiene, β-phellandrene, α-terpineol, 1R-α-pinene, and β-caryophyllene] were exclusively identified in control fruits, whereas 9 were found in *LcTPS24*-silenced fruits [β-patchoulene, himachala-3(12),4-diene, (+)-cyclosativene, α-amorphene, β-gurjunene, δ-cadinene, α-cubebene, (-)-α-thujene, and 4-δ-carene], with 12 compounds being common (Figure 6E). Furthermore, the heatmap analysis revealed distinct emission patterns for different compounds in both control and *LcTPS24*-silenced fruits (Figure 6F). In short, a distinct variation in terpene emission was observed before and after silencing *LcTPS24* in litchi fruits.

## 4. Discussion

Fruit aroma is a complex quality trait arising from the composition, abundance, and sensory properties of volatile organic compounds (VOCs). In litchi, understanding the biochemical processes underlying its characteristic floral–fruity aroma requires both characterization of the volatile mixture and functional investigation of the enzymes involved in its production. The cultivar-level profiles obtained in this study indicate that terpenes constitute a substantial component of the detected VOCs, supporting further investigation of terpene metabolism as a contributor to variation in fruit volatile composition. This finding is consistent with previous reports implicating monoterpenes, including citronellol, geraniol, myrcene, and limonene, in litchi aroma [11,12,18]. However, the sensory importance of individual compounds cannot be inferred directly from their relative abundance, because odor thresholds and interactions among volatile constituents also influence aroma perception.

The differences in monoterpene and sesquiterpene proportions among cultivars suggest variation in the processes governing terpene production and accumulation. Such differences may reflect variation in TPS expression and activity, precursor availability, or subsequent metabolite conversion, acting in conjunction with developmental changes. The stage-specific profiles also indicate that volatile composition does not follow a uniform trajectory during ripening; consequently, cultivar-level summaries aggregated across S1–S3 should not be interpreted as evidence of a consistent increase in terpene abundance toward maturity. Although cultivar-associated volatile patterns may provide candidates for chemical discrimination, their suitability as biomarkers requires validation using independent samples.

Within this context, LcTPS24 was selected for functional investigation on the basis of our previous characterization of the litchi TPS family. Its elevated expression during early pericarp development and positive associations with monoterpenes provided a rationale for candidate selection, rather than evidence that it was the dominant determinant of fruit aroma. The conserved sequence features and phylogenetic placement of LcTPS24 further supported its annotation as a TPS candidate. In particular, the C-terminal DDxxD and NSE/DTE motifs are associated with metal-ion coordination and substrate ionization, whereas the N-terminal RRx_8_W motif represents a distinct conserved feature found in many monoterpene synthases [25,26,27]. Its placement within TPS-b is consistent with the biochemical activity observed in this study, but neither subfamily membership nor motif conservation independently establishes product specificity, catalytic efficiency, or subcellular localization.

The expression profiles provide additional biological context by indicating that LcTPS24 transcript accumulation varies among tissues, cultivars, and developmental stages (Figure 4). The higher plotted expression in peel and the cultivar-dependent timing of expression maxima suggest that its contribution to terpene metabolism may differ during fruit development. Nevertheless, transcript abundance cannot be assumed to correspond directly to metabolite abundance, because protein accumulation, substrate availability, enzyme activity, and metabolite turnover can influence the final volatile profile. Furthermore, the separate peel and aril expression measurements cannot be directly matched to the combined-tissue VOC profiles to establish tissue-specific relationships. These observations therefore support investigation of a developmental role for LcTPS24 without demonstrating that its transcriptional variation alone explains differences in volatile composition among cultivars.

The recombinant enzyme assays provide evidence that LcTPS24 supports GPP-dependent formation of multiple putatively assigned monoterpenes (Figure 5). The generation of several products from a single substrate is consistent with the catalytic versatility of plant terpene synthases, in which alternative rearrangements and termination reactions can produce structurally related compounds [28,29,30]. This reaction chemistry offers a plausible framework for interpreting the observed product profile, although the intermediates and active-site residues responsible for product branching have not been established for LcTPS24. The reported peak-area distribution suggests unequal contributions of individual products, but definitive assignments and proportions require compound-specific analytical validation. In addition, relative product proportions describe reaction output rather than catalytic efficiency; the absence of kinetic measurements precludes conclusions concerning substrate affinity, turnover, or comparative enzyme performance.

Product diversity must also be distinguished from substrate specificity. LcTPS24-dependent product formation was detected with GPP, whereas no corresponding activity was detected with FPP under the conditions tested. These findings support its characterization as a GPP-dependent monoterpene synthase in the present assays, but do not establish absolute substrate exclusivity under other conditions. Similarly, GPP utilization and TPS-b membership are compatible with a potential role in monoterpene biosynthesis supplied by plastidial precursors, but they do not demonstrate plastid localization or exclusive dependence on the MEP pathway. The pathway illustrated in Figure 5C should therefore be regarded as a proposed biosynthetic context. Experimental localization and precursor-tracing studies would be needed to determine the intracellular site of LcTPS24 activity and the origin of its substrate supply.

The VIGS experiment provides complementary evidence for a contribution of LcTPS24 to fruit monoterpene metabolism. In JGHN fruit, reduced *LcTPS24* transcript abundance was accompanied by lower monoterpene accumulation, a response consistent with the recombinant enzyme activity (Figure 6). However, attribution of this response specifically to *LcTPS24* depends on the specificity of the silencing fragment and the reliability of RT–qPCR normalization. The accompanying decline in sesquiterpenes cannot be explained by demonstrated direct FPP conversion by LcTPS24, because this activity was not detected in vitro. Altered precursor allocation, coordinated responses of other biosynthetic genes, and unintended silencing of related TPSs remain possible explanations that have not been distinguished experimentally. Off-target assessment, expression analysis of closely related TPS genes, and complementary gain-of-function or rescue experiments would strengthen the causal interpretation. Because silencing was examined in JGHN, the results also cannot establish equivalent functional effects across the other cultivars.

Electronic-tongue measurements offer a separate description of treatment-associated variation in juice sensor responses and should be interpreted independently of the GC–MS profiles. Specifically, the PCA in Figure 6D represents electronic-tongue responses rather than volatile terpene composition, while the Venn diagram and heatmap summarize compound detections and VOC patterns rather than compound-level correlations. The concurrent differences between treatments permit a descriptive comparison, but the absence of paired numerical datasets prevents quantitative assessment of relationships between individual volatiles and sensor responses. These measurements therefore do not demonstrate that altered terpene accumulation caused changes in taste or perceived aroma. Direct sensory evaluation, GC–olfactometry, and appropriately calibrated odor activity estimates would be required to establish the sensory significance of the observed metabolic changes.

Overall, the combination of recombinant enzyme activity and silencing-associated changes supports a contribution of LcTPS24 to monoterpene biosynthesis in litchi fruit. The principal limitations concern compound-identification confidence, the absence of kinetic characterization, unresolved silencing specificity and reference-gene stability, and the lack of direct sensory evidence. These limitations define the scope of the current interpretation: the study advances the functional characterization of a selected TPS candidate, while its relative contribution within the TPS family, the regulation of its expression, and its influence on characteristic fruit aroma remain to be established.

## 5. Conclusions

This study characterized LcTPS24 as a terpene synthase exhibiting GPP-dependent, multiproduct monoterpene synthase activity under the tested in vitro conditions. Silencing of *LcTPS24* in JGHN fruit was accompanied by reduced monoterpene accumulation, providing complementary evidence consistent with a contribution to fruit terpene biosynthesis. The cultivar and developmental profiles provide a broader context for this function, but do not establish LcTPS24 as the dominant determinant of volatile variation or characteristic litchi aroma. Likewise, the electronic-tongue results describe treatment-associated differences in juice sensor responses and do not directly demonstrate altered aroma perception. These findings provide a basis for further investigation of LcTPS24 within the litchi TPS family, with additional analytical validation, assessment of gene-specific effects, and direct sensory evaluation needed to determine its relevance to fruit flavor improvement.

## Data Availability

The original contributions presented in this study are included in the article. Further inquiries can be directed to the corresponding authors.
